# Muscle Characteristics in Pediatric Hereditary Spastic Paraplegia vs. Bilateral Spastic Cerebral Palsy: An Exploratory Study

**DOI:** 10.3389/fneur.2021.635032

**Published:** 2021-02-26

**Authors:** Nathalie De Beukelaer, Lynn Bar-On, Britta Hanssen, Nicky Peeters, Sandra Prinsen, Els Ortibus, Kaat Desloovere, Anja Van Campenhout

**Affiliations:** ^1^KU Leuven Department of Rehabilitation Sciences, Leuven, Belgium; ^2^Clinical Motion Analysis Laboratory, University Hospitals Leuven, Leuven, Belgium; ^3^Department of Rehabilitation Medicine, Amsterdam University Medical Center, Amsterdam, Netherlands; ^4^Department of Orthopedics, University Hospitals Leuven, Leuven, Belgium; ^5^KU Leuven Department of Development and Regeneration, Leuven, Belgium

**Keywords:** hereditary spastic paraplegia, cerebral palsy, instrumented impairment assessments, ultrasound, muscle morphology, muscle volume, hyperreflexia, spasticity

## Abstract

Hereditary spastic paraplegia (HSP) is a neurological, genetic disorder that predominantly presents with lower limb spasticity and muscle weakness. Pediatric pure HSP types with infancy or childhood symptom onset resemble in clinical presentation to children with bilateral spastic cerebral palsy (SCP). Hence, treatment approaches in these patient groups are analogous. Altered muscle characteristics, including reduced medial gastrocnemius (MG) muscle growth and hyperreflexia have been quantified in children with SCP, using 3D-freehand ultrasound (3DfUS) and instrumented assessments of hyperreflexia, respectively. However, these muscle data have not yet been studied in children with HSP. Therefore, we aimed to explore these MG muscle characteristics in HSP and to test the hypothesis that these data differ from those of children with SCP and typically developing (TD) children. A total of 41 children were retrospectively enrolled including (1) nine children with HSP (ages of 9–17 years with gross motor function levels I and II), (2) 17 age-and severity-matched SCP children, and (3) 15 age-matched typically developing children (TD). Clinically, children with HSP showed significantly increased presence and severity of ankle clonus compared with SCP (*p* = 0.009). Compared with TD, both HSP and SCP had significantly smaller MG muscle volume normalized to body mass (*p* ≤ 0.001). Hyperreflexia did not significantly differ between the HSP and SCP group. In addition to the observed pathological muscle activity for both the low-velocity and the change in high-velocity and low-velocity stretches in the two groups, children with HSP tended to present higher muscle activity in response to increased stretch velocity compared with those with SCP. This exploratory study is the first to reveal MG muscle volume deficits in children with HSP. Moreover, high-velocity-dependent hyperreflexia and ankle clonus is observed in children with HSP. Instrumented impairment assessments suggested similar altered MG muscle characteristics in pure HSP type with pediatric onset compared to bilateral SCP. This finding needs to be confirmed in larger sample sizes. Hence, the study results might indicate analogous treatment approaches in these two patient groups.

## Introduction

Hereditary spastic paraplegia (HSP) is described as a genetic, heterogeneous disorder leading to axonal degeneration of the spinal pathways and mostly classified in either pure or complex (i.e., additional symptoms rather than pyramidal signs) types (1, 2). In children with the pure HSP type presenting symptoms already from infancy or childhood, bilateral spasticity and muscle weakness in the lower limbs contribute to gait deviations (3, 4). This predominant clinical picture is similarly observed in children with bilateral spastic cerebral palsy (SCP) (2, 4). Hence, the symptomatic treatment management in HSP is often analogous to the common treatment in SCP. Conservative treatment includes orthotic management and regular physiotherapy, which incorporates strengthening, stretching, and gait rehabilitation. This approach is commonly combined with spasticity management [i.e., oral medication, intrathecal baclofen therapy, and/or intramuscular botulinum neurotoxin (BTX) injections; (5–7)].

Caused by upper motor neuron lesions in the developing brain, children with SCP present with neuromotor symptoms already within the first 2 years of life. Therefore, these children have an atypical development resulting in altered muscle function and pathological gait (5). In the last decade, there has been growing research interest in the muscle morphology of children with SCP, with studies showing evidence for decreased muscle volume (MV) and muscle belly length of the medial gastrocnemius (MG) muscle in comparison with typically developing (TD) children (8). The calf muscle is most often selected as a target muscle because of its pathological involvement in the clinical presentation and consequently functional importance (9). Muscle morphology is considered related to muscle function in the sense that decreased MV has been indicated as one of the major contributors to muscle weakness (10, 11). Moreover, altered MG muscle morphology combined with the manifestation of lower limb spasticity has been observed already from early ages (12, 13).

Muscle imaging using 3D-freehand ultrasound (3DfUS) is a reliable and valid method to determine macroscopic alterations of muscle morphology in children with SCP (14–16). In addition, instrumented assessment of hyperreflexia provides reliable, objective parameters to evaluate the stretch of hyperreflexia by combining surface electromyography (sEMG) signals with joint motion analysis during manually applied muscle stretches at low and high velocity (17, 18). This quantitative approach enables the assessment of the neural component of muscle hyper-resistance, which is further indicated as hyperreflexia (19). Moreover, using such instrumentation, different muscle activation patterns in reaction to increased stretch velocity have been reported (20). Treatment response may differ between these phenotypes, highlighting the importance to use instrumented impairment assessment for treatment guidance (21, 22).

In more than half of the children who are clinically suspected of having HSP, the genetic diagnosis is not confirmed by means of whole exome sequencing or HSP gene panels (23). The latter considers the pathogenic mutations in the spastic paraplegia genes (SPG) known to date (24). Moreover, since the clinical picture is often suggestive of SCP, other markers for a good differential diagnosis are a priority (2). Previous research has already pointed out possible indications for the HSP condition based on the gait deviations, whereby these results could contribute to the diagnostic process of children with HSP (25, 26). In the same sense, an objective and quantitative investigation of both hyperreflexia and potential alterations in muscle morphology in children with HSP might help to differentiate them from SCP.

Whereas, the clinical presentation indicates an overlap in children with HSP and SCP, it is unclear if similarities are also presented in terms of muscle characteristics. These insights might help to distinguish HSP and SCP and, thus, potentially provide markers for more accurate diagnosis, which may support the treatment management in the HSP population and optimize treatment plans toward delineated patient-specific approaches. However, to date, muscle characteristics in children with HSP have not yet been studied.

Therefore, the first aim of this retrospective study is to explore the MG muscle characteristics in children with HSP using instrumented impairment assessments. Second, we tested the hypothesis that the muscle morphology and hyperreflexia in children with HSP differ from those of children with SCP.

## Materials and Methods

### Participants

This study retrospectively selected 41 participants who were recruited via the Clinical Motion Analysis Laboratory in collaboration with the pediatric–orthopedic department of the University Hospitals Leuven. Children underwent one or more assessments including clinical examination, 3DfUS and instrumented assessment of hyperreflexia. Written informed consent was signed by all parents of the children. The study assessments were performed as part of different ongoing research projects and were approved by the Ethical Committee of the University Hospitals Leuven and KU Leuven (S56977 and S59945).

In the previous described research projects, children with a genetically confirmed diagnosis of HSP were eligible for inclusion. In the absence of a genetic diagnosis after performing extensive genetic workup, a clinical HSP diagnosis was made by qualified professionals based on (a) neurological findings, such as spasticity and weakness in the plantar flexor muscles, (b) the age of onset, and if available, (c) a positive family history of gait disturbances, and (d) normal neuro-imaging, electromyography measures, and metabolic investigations [Supplementary Table 1; (1, 2, 27)]. The latter is performed to ensure the exclusion of other disorders (2, 28). As a result, from the population of children with HSP who received regular clinical follow-up between May 2017 and July 2020 at the University Hospitals Leuven, 17 patients were assessed as part of ongoing research projects (S56977 and S59945).

**Figure 1:**
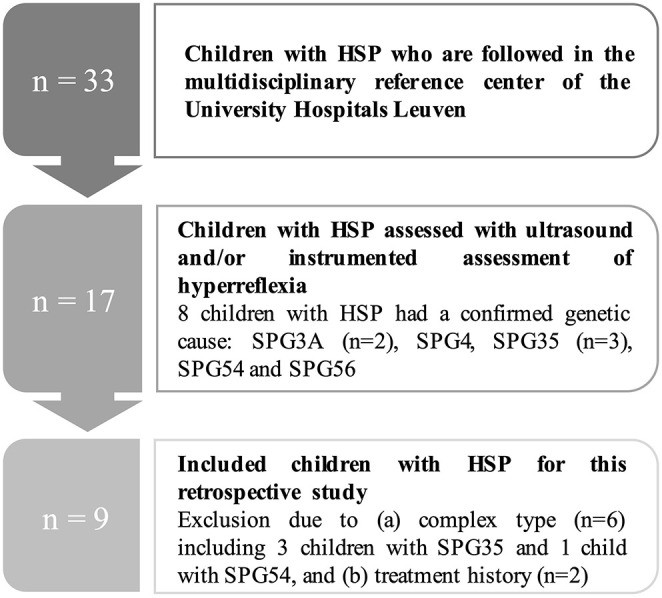
Flowchart of the patient selection process for the children with hereditary spastic paraplegia (HSP). *N*, number.

For this retrospective study, the following inclusion criteria for children with either clinically or genetically confirmed HSP were applied: (a) aged between 8 and 17 years; (b) level I or II on the gross motor function classification system (GMFCS); (c) uncomplicated or pure type of HSP; (d) no BTX injections in the previous 6 months; (e) no orthopedic surgery in the previous 2 years; (f) no history of soft-tissue musculoskeletal- or neurosurgery (e.g., selective dorsal rhizotomy), and (g) no intrathecal baclofen pump. This resulted in a group of nine children with HSP, including six boys and three girls. Mutations in SP-designated genes were confirmed in four patients (two children with SPG3A, one with SPG4, and one with SPG56). For the other five participants, genetic analysis could not indicate mutations in the SP-designated genes. The flowchart of the HSP patient selection and inclusion process is presented in Figure 1.

Based on the age, GMFCS levels and gender of this HSP group, a cohort of children with bilateral SCP were group matched. These children were selected from a retrospective database (S59945) that was established during an ongoing research project on muscle characteristics in children with SCP. To aim for a maximum number of children that were eligible from the retrospective database, group matching was performed per available assessment. As a result, 15 children with SCP were included with 3DfUS data, and nine children with SCP were included with hyperreflexia data. Seven children with SCP were measured for both assessments. Supplementary Figure 1 gives a schematic overview of the study samples per assessment. To confirm the diagnosis of bilateral SCP, these children showed abnormal neuro-imaging and the clinical presentation of plantar flexor muscle spasticity.

The muscle morphology of these two patient groups was compared with a reference group of 15 TD children, which was retrospectively included based on the available 3DfUS data in the Clinical Motion Analysis Laboratory database (S59945). This cohort was group matched following the age and gender of the HSP group. No reference of instrumently assessed hyperreflexia data were used for the TD group, since the muscle activation during passive muscle stretches in TD children is negligible (17).

### Data Collection and Processing

Assessments were carried out by the different experienced examiners who were involved in the research projects, whereas all data processing was performed by the same experienced researcher (ND). As defined by the clinical examination, the most affected leg was measured in the children with HSP and SCP, whereas a random leg was selected in the TD children. Anthropometric data including body mass, body length, and leg lengths were measured. The latter was measured from the lower border of the anterior superior iliac spine to the lower border of the medial malleolus.

### Clinical Assessments

Clinical examination was performed including the passive range of motion to maximal ankle dorsiflexion (ROM), plantarflexor spasticity assessed with the Modified Ashworth Scale (MAS) and Modified Tardieu Angle (MTA), and plantarflexor strength using the Medical Research Council grade scale (29–31). ROM, MAS, and MTA were measured with the knee extended. The presence and severity of the clonus was clinically graded based on how quickly a rhythmic, oscillating reflex appears, and disappears while stretching the ankle towards the dorsiflexion position. The clonus grading is as follows: 0, no clonus; 1, clonus after multiple stretches; 2, quick clonus, quick stop; 3, quick clonus, slow stop, and 4, quick clonus, no stop. Clinical scores including the Functional Mobility Scale (FMS), walking ability, self-selected gait speed, presence of pain during gait, and bladder and bowel function were retained from the clinical assessments, which were performed at the time of or within 1 year of the study assessment, as part of the routine clinical follow-up. The FMS describes the functional mobility by scoring the walking ability over three distances (i.e., 5–50–500 m) on a six-point scale for which lower scores indicate more need for assistance (32).

### 3D-Freehand Ultrasound

To aim for a comfortable and resting joint position, the children were positioned in prone with a triangular cushion placed under the lower leg whereby the ankle was placed over the edge of this cushion. This resting position provided ~25° of knee flexion and 30° of plantar flexion. Muscle morphology was assessed using 3DfUS, combining a 2D ultrasound system and motion-tracking system of three fixed optical cameras, with the same instrumentation settings as described by Schless et al. (33). US images of the MG muscle were acquired in a transverse orientation, starting from the medial femoral condyle to the distal end of the calcaneus. Both data collection and processing were completed using STRADWIN software (version 6.0; Mechanical Engineering, Cambridge University, Cambridge, UK). The following processing guidelines to assign landmarks in the transverse plane were applied: (a) MG muscle origin was defined as the most superficial part of the medial femoral condyle, (b) the muscle–tendon junction (MTJ) was defined as the first image after the last image with visible muscle belly mass, and (c) the first image of the calcaneus bone was defined as the tendon insertion. Data processing started with segmenting the created 3D-reconstruction by manually outlining the cross-sectional areas along the inside of the muscle borders starting from the defined origin to the last image before the MTJ. A linear interpolation between the outlined borders was applied to compute the MV (in ml). In addition, MV was normalized to body mass (ml/kg). Muscle belly lengths (ML) were defined from the origin to the MTJ, and the tendon lengths (TL) were defined from the MTJ to the tendon insertion. The distance between the muscle origin and insertion was specified as the muscle–tendon unit complex length (MTUL). The same assessor assigned the landmarks twice, and the average of the length scores was calculated. ML, TL, and MTUL data were reported in millimeter (mm). Both ML and MTU L were normalized for leg length (i.e., nML and nMTUL) and therefore expressed as percentages.

### Instrumented Assessment of Hyperreflexia

A previously described measurement protocol was applied in which muscle activation and joint motion analysis data were simultaneously collected during manually applied ankle rotations to stretch the MG muscle at low (LV) and high (HV) velocity (17). Muscle activity was registered using sEMG data (Zerowire, Cometa, Milan, IT) whereby circular Ag/Ag Cl electrodes (diameter 2 cm) were attached on the MG and tibialis anterior muscle bellies following the SENIAM guidelines (34). Inertial measurement units were placed on the shank and foot to analyze the ankle joint motions. Participants were assessed in supine position on an examination table, while the lower leg rested on a support to avoid contact of the MG muscle sEMG sensors with the table. The foot was fixated in a custom-made orthotic with an attached force sensor. Data of the latter were not reported in the current study. While holding on the force sensor with the shank in a fixed position, the examiner manually rotated the ankle from maximal plantarflexion to maximal dorsiflexion position. Four passive rotations over the full ankle ROM were performed at LV (i.e., in 5 s) and then four rotations were repeated at HV (i.e., performed as fast as possible). During each velocity trial, the participants were asked to remain relaxed, and a rest period of 7 s between the four repetitions was taken to minimize post-activation depression.

Offline data processing was performed using a custom-made Matlab software (Mathworks, R2017b). Data quality checks for velocity consistency and quality of the EMG signals were performed as described by Bar-On et al. (17). ROM and the maximum angular velocity (V_max_) were calculated and averaged over the repetitions per velocity trial. Hyperreflexia parameters were based on the registered MG muscle activity whereby first, the raw sEMG data were filtered, and second, root-mean square (RMS)-EMG (μV) was calculated, as previously described for both steps (17). Hyperreflexia was defined as the average RMS-EMG over the duration of the maximal velocity zone. This time zone was restricted to 200 ms prior to Vmax up to 90% of the full ROM. In addition, this hyperreflexia parameter was normalized to the peak RMS-EMG value of the obtained maximum voluntary isometric contractions (MVIC) and expressed as percentage. Both non-normalized and normalized hyperreflexia parameters were averaged over the stretch repetitions for the LV trial as well as for the change between the high and low velocity trials (change HV–LV). The latter is important to investigate the velocity-dependent nature of muscle hyperreflexia. In addition, visualization of normalized sEMG data across the LV and HV stretches over the ROM were used to classify responses according to previously described muscle activation patterns (i.e., velocity- or length-dependent pattern). More details on the definition of hyperreflexia parameters and muscle activation patterns can be found in earlier publications (17, 20).

### Statistical Analysis

Statistical analyses were performed using SPSS (IBM SPPS Statistics version 27), and graphs were designed in GraphPad Prism 9 and Matlab software. Normality of the data was both visually checked and tested with the Shapiro–Wilk test. Due to the small sample size and non-normally distributed data, descriptive statistics included median and interquartile ranges and non-parametric test were applied. Independent-sample Kruskal–Wallis test was carried out to explore differences in mean ranks of age, anthropometric data, and muscle morphology between the three groups. Group differences were tested with *post-hoc* Mann–Whitney *U* (MWU) test. Bonferroni adjustment for multiple testing was applied after these *post-hoc* MWU analyses (*p* < 0.017). Comparisons of the clinical examination data, performance, and hyperreflexia parameters between the HSP and SCP groups were performed with MWU analyses, whereby the level of significance was set at *p* < 0.05.

## Results

Participant characteristics and anthropometric data of the nine children in the HSP group were compared to (a) data of 15 participants in the SCP group and 15 participants of the TD group defined by 3DfUS, and (b) data of nine participants in the SCP group, defined by instrumented assessment of hyperreflexia (Table 1). No differences were found between the groups for age, body mass, body length, and leg length. Results of the clinical examination for ankle ROM, spasticity, and muscle strength did not differ significantly between the HSP group (*n* = 8, missing data for one child) and all children with SCP (*n* = 17) (Table 2). For the HSP group (*n* = 8), only one child had low MAS-scores (MAS 1+) and seven children had high MAS-scores (four MAS of 2; three MAS of 3), whereas in the SCP group (*n* = 17), 10 children had low MAS-scores (two MAS 1 and eight MAS 1+) and seven children had high MAS-scores (two MAS 2; five MAS 3). The ankle clonus scores differed significantly between the HSP and the total SCP group (*p* < 0.009). Specifically, only one child with HSP did not present a clonus, whereas the majority of children with SCP (i.e., 11 out of 17) had no clonus. All children in the HSP and SCP groups had regular physiotherapy with a median of 120 min per week and 100 min per week, respectively. One third of the HSP children frequently used day orthoses, whereas 10 out of the 17 children with SCP used orthoses during the day. For both groups, more than 50% of the children were never administered with BTX injections (Supplementary Table 2). The HSP and SCP cohort presented with median FMS scores of 6-6-5 and 6-5-5, respectively. More than half of the children in both groups were able to walk for more than 3,000 m, whereas the self-selected walking speed was slightly or even moderately reduced. Especially in the SCP group, 12 children out of 17 presented with a walking speed of more than 10 s over a 10-m distance. Pain during gait was reported by only two and three children in the HSP and SCP groups, respectively (Supplementary Table 3).

**Table 1:** Participant characteristics and anthropometric data in the HSP, SCP, and TD group.

	HSP*^†^ (*n* = 9)	SCP* (*n* = 15)	**SCP^**†**^ (*n* = 9)**	TD* (*n* = 15)	p*	***p*^**†**^**
Age (y)	12.9 (5.5)	11.3 (5.8)	11.2 (5.9)	13.8 (5.6)	0.935	0.340
GMFCS level (n)	I = 4, II = 5	I = 7, II = 8	I = 4, II = 5	NA		
Gender (G/B)	3/6	6/9	3/6	6/9		
Body mass (kg)	47.0 (18.6)	44.5 (27.5)	38.5 (2.6)	50.4 (25.3)	0.652	0.136
Body length (cm)	159.5 (27.6)	149.0 (25.6)	146.6 (27.6)	164.5 (32.4)	0.491	0.387
Leg length (cm)	83.5 (12.7)	75.7 (16.4)	75.5 (16.3)	86.2 (18.2)	0.201	0.436

*Data are shown as median (interquartile range)*.

* and † indicated the different group comparisons. HSP, hereditary spastic paraplegia; SCP, spastic cerebral palsy; TD, typically developing; n, number; y, years; GMFCS, gross motor function classification system; NA, not applicable; G, girl; B, boy.

**Table 2:** Median (minimum and maximum values) of all clinical examination results in the HSP and SCP group.

	**HSP (*n* = 8)**	**SCP (*n* = 17)**	**HSP-SCP, *p***
ROM (°)	5 (−5; 10)	5 (−5; 15)	1.00
MAS	2 (1.5; 3)	1.5 (1; 3)	0.140
MTA (°)	−13 (−30; 5)	−10 (−30; 0)	0.636
MRC	4 (3; 4)	3 (2; 5)	0.549
Clonus	2 (0; 4)	0 (0; 2)	0.009

*Missing data of one child in the HSP group. HSP, hereditary spastic paraplegia; SCP, spastic cerebral palsy; n, number; ROM, range of motion to maximal ankle dorsiflexion; MAS, Modified Ashworth Scale; MTA, Modified Tardieu Angle; MRC, Medical Research Council*.

Compared with the TD reference group, both children with HSP and SCP differed significantly in the MG muscle volume normalized to the body mass (*p* ≤ 0.001) with the greatest deficits in the SCP group, namely, 32%. *Post-hoc* MWU test revealed only a significantly smaller absolute MV in the children with SCP compared with TD children (*p* = 0.004; Supplementary Figure 2). Muscle lengths were not significantly different between the three groups, although normalized muscle belly lengths (i.e., ML/MTUL and nML) tended to be lower in both patient groups compared with the TD group, with more alterations in the SCP group (Table 3).

**Table 3:** Median (and IQR) of medial gastrocnemius muscle morphology outcomes in the HSP, SCP, and TD group.

	**HSP (*n* = 9)**	**SCP (*n* = 15)**	**TD (*n* = 15)**	***p***	**HSP-SCP, *p***	**HSP-TD, *p***	**SCP-TD, *p***
MV (ml)	80.0 (51.7)	73.0 (29.1)	127.3 (60.4)	0.017	1.00	0.084	0.004
nMV (ml/kg)	1.8 (0.4)	1.7 (0.7)	2.6 (0.4)	0.001	1.00	0.001	0.001
ML (mm)	199.6 (36.7)	185.3 (30.1)	213.5 (51.5)	0.078			
TL (mm)	170.2 (49.2)	173.1 (39.3)	169.8 (39.7)	0.978			
MTUL (mm)	390.2 (73.4)	363.8 (51.4)	388.3 (87.7)	0.237			
ML/MTUL (%)	54.0 (1.0)	53.0 (4.0)	57 (1.0)	0.141			
nML (%)	24.7 (3.9)	24.4 (2.5)	26.1 (4.2)	0.101			
nMTUL (%)	45.9 (2.5)	45.9 (3.0)	46.4 (1.2)	0.683			

*HSP, hereditary spastic paraplegia; SCP, spastic cerebral palsy; TD, typically developing; n, number; MV, muscle volume; nMV, muscle volume normalized to body mass; ML, muscle belly length; TL, tendon length; MTUL, muscle-tendon unit complex length; nML, ML normalized to leg length; nMTUL, MTUL normalized to leg length*.

**Figure 2:**
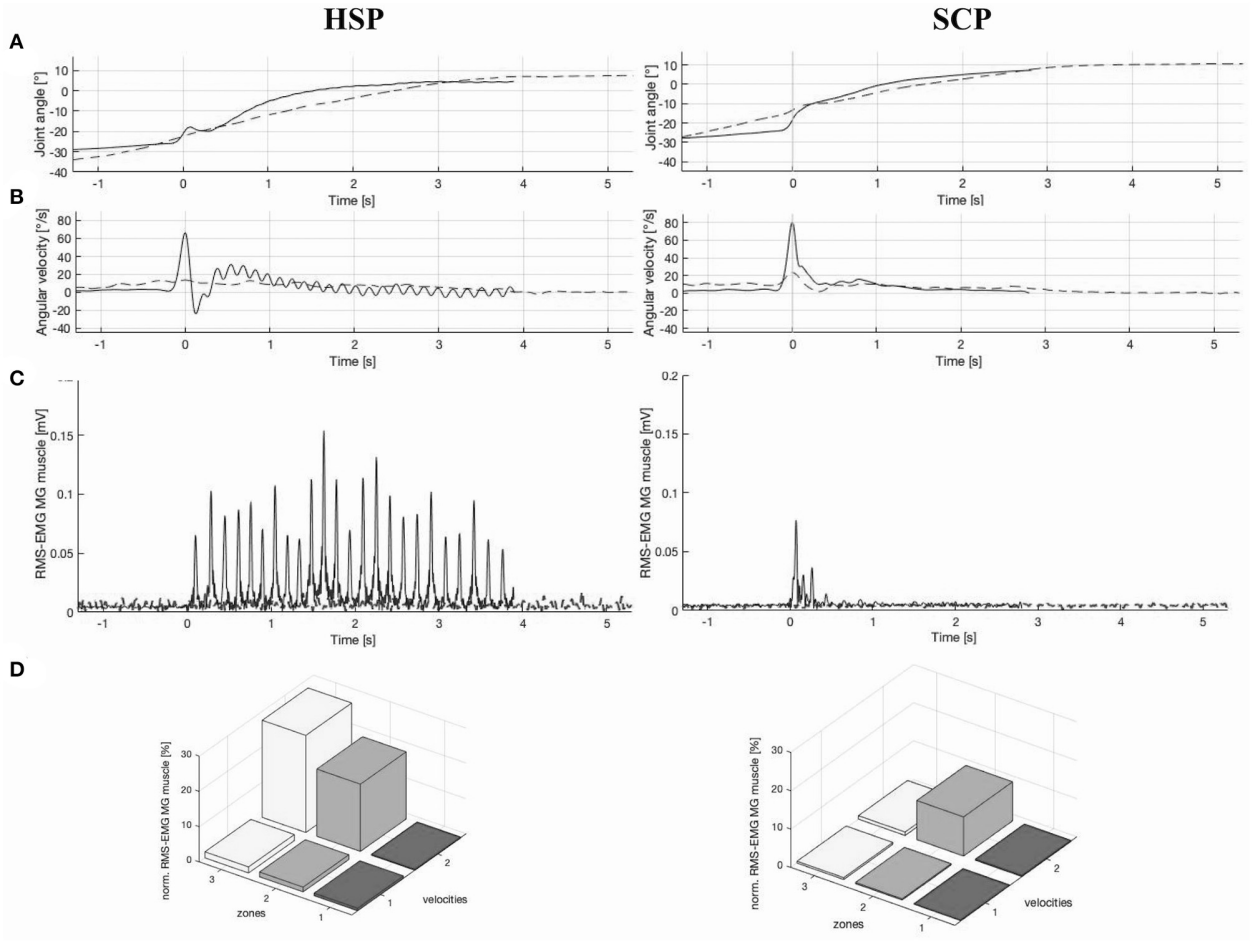
Example illustration of **(A)** ankle joint angle vs. time graph, **(B)** ankle angular velocity vs. time graph, **(C)** root-mean square-electromyography (RMS-EMG) vs. time graph, and **(D)** 3D bar graph of average normalized RMS-EMG across three position zones and the two velocities of the medial gastrocnemius (MG) muscle, in a child with hereditary spastic paraplegia (HSP) and an age-matched child with spastic cerebral palsy (SCP) (20). Graphs **(A–C)** presented both a slow (dashed line) and high (continuous line) velocity stretch, whereby 0 s represent the time at the maximal velocity. In addition, the RMS-EMG vs. time graph **(C)** for the child with HSP indicates a clear ankle clonus, with 24 oscillations over a time period of 4 s. For the SCP case, this graph **(C)** indicates a catch during the high velocity stretch. In graph **(D)**, the position zones indicate 10–90% of range of motion (ROM) divided in three equal parts. Velocity 1 and velocity 2 represent the low-velocity and high-velocity muscle stretches, respectively. High-velocity-dependent activation patterns are presented in both the HSP and SCP case.

Ankle joint rotations during HV stretch were performed over a median ROM of 50.7° (15.1) and 52.8° (18.4) with median maximal velocity of 81.1°/s (88.4) and 122.9°/s (45.6) in the HSP and SCP groups, respectively. The between-group differences in ankle joint rotations were not significant. Both non-normalized and normalized hyperreflexia parameters for LV and for the change between HV and LV were not significantly different between the HSP and SCP groups (Table 4). Higher hyperreflexia values, especially for change in HV–LV, tended to be present in the HSP group, compared with the SCP group. Eight children with HSP were categorized in a pure high-velocity-dependent pattern, whereas this pattern was observed in only six children with SCP. Additionally, muscle activity in one child of the HSP and three children of the SCP group was categorized as mixed high-velocity-dependent activation pattern. Figure 2 shows an example joint motion and muscle activation data during the instrumented assessment of hyperreflexia in a child with HSP and age-matched child with bilateral SCP.

**Table 4:** Median (and IQR) of performance- and hyperreflexia-parameters in the HSP and SCP group.

	**HSP (*n* = 9)**	**SCP (*n* = 9)**	**HSP-SCP, *p***
ROM, LV (°)	50.7 (15.1)	52.8 (18.4)	0.796
ROM, HV (°)	49.6 (12.4)	52.0 (22.4)	1.00
V_max_, LV (°/s)	17.4 (7.6)	20.5 (12.3)	0.666
V_max_, HV (°/s)	81.1 (88.4)	122.9 (45.6)	0.387
MVIC (mV)	0.2 (0.1)	0.2 (0.2)	0.666
RMS-EMG, LV (μV)	1.0 (0.9)	1.1 (2.1)	0.546
Norm. RMS-EMG, LV (%)	1.0 (1.0)	0.5 (2.3)	0.340
RMS-EMG, HV-LV (μV)	9.1 (9.6)	4.5 (7.2)	0.113
Norm. RMS-EMG, HV-LV (%)	7.9 (9.6)	3.7 (7.3)	0.222

*HSP, hereditary spastic paraplegia; SCP, spastic cerebral palsy; n, number; ROM, range of motion; LV, low velocity; HV, high velocity; V_max_, maximal angular velocity; MVIC, maximal voluntary isometric contraction; Norm., normalized*.

## Discussion

The purpose of this exploratory study was to investigate whether the MG exhibits muscle-related differences in two patient groups who are known to present with similar clinical impairments, despite their different etiology. To date, HSP is unexplored in terms of muscle morphology and instrumented assessment of hyperreflexia. In this study, only children presenting the pure or uncomplicated HSP type (i.e., solely pyramidal signs) with a pediatric onset were retrospectively included. This restriction minimized the possibility of a progressive disease course as seen in complicated and late onset HSP types (35). In the HSP group of this study, the mean age of symptom onset was 2.7 years, ranging from 2 months to 9 years old (Supplementary Table 1). The confirmed gene mutations in three of the four patients are described as the most common cause of autosomal dominant HSP. The mutations in these SPG3A and SPG4 cases and their HSP-associated proteins atlastin-1 and spastin, respectively, result in dysfunctional axonal transport with consequently corticospinal motor neuron degeneration (2, 36–38). Moreover, children with SPG3A and SPG4 are known to present similarities in upper motor neuron signs that are commonly observed in children with bilateral SCP (39). In line with the current literature, the included child with the SPG56 subtype, which is described as recessive–autosomal HSP, is presenting as pure form with an early onset [i.e., at the age of 2 years; (40, 41)]. The involved gene mutation results in altered mitochondrial architecture and function (42). To further ensure differences in etiology between the two groups, only children with SCP showing clear, abnormal neuro-imaging findings (e.g., periventricular leukomalacia) were included (43). In the current study, the results of the clinical examination showed comparable levels of ankle mobility, spasticity, and muscle strength between both patient groups. However, the ankle clonus score was significantly higher in children with HSP compared with those with SCP, indicating increased presence and severity with mostly a quick clonus appearance with either slow or no stop (Supplementary Table 4).

### Muscle Morphology

Altered MG muscle morphology in children with HSP was demonstrated by significantly smaller MV normalized to body mass of 30%, compared to TD. Furthermore, no significant differences were observed between the HSP and SCP groups, which indicate similarities in altered MG muscle morphology. Although the children in the SCP group tended to be lower in body mass and shorter in length than the children in the HSP group, normalization to this anthropometric data did not reveal significant differences in muscle volumes, nor in muscle lengths between both groups. However, a trend towards longer muscle belly lengths normalized to leg length was observed in the HSP group compared with the SCP group.

In agreement with previous research, muscle volumes in children with SCP were significantly smaller than the muscle volumes of the TD reference group (8). In the current study, average muscle volume deficits of 21% in the HSP group and 35% in the SCP group were observed when compared with the TD data. Interestingly, these differences are larger than the previously reported minimal detectable changes of 9.9% for MG muscle volumes (15). In the same sense, Noble et al. previously reported deficits of 38% in MG muscle volume for children with bilateral CP, with the same age ranges (i.e., older than 9 years) and similar levels of ambulatory ability [i.e., GMFCS levels I and II; (44)]. No significant differences were found with respect to the muscle lengths. Nonetheless, in comparison with data of the TD peers, absolute muscle belly lengths were 4 and 12% shorter in the HSP and SCP groups, respectively. These results were in line with previous research reporting 10% shorter muscle lengths in the paretic limb of children with unilateral SCP compared with data of TD children (45). However, only limited 3D ultrasound data of MG muscle lengths in children older than age 12 years have been reported so far (9). In conclusion, altered muscle volumes were observed in children with HSP and showed similarities to the muscle alterations in children with SCP.

### Hyperreflexia

Hyperreflexia parameters were not significantly different between the HSP and SCP groups. Pathological muscle activity with increasing stretch velocity was observed in both patient groups, with a tendency of more hyperreflexia in the HSP group. This velocity-dependent hyperreflexia was confirmed by the results of the classification in hyperreflexia muscle activation patterns. Most of the children with HSP were classified with pure high-velocity-dependent muscle activation pattern. The amount of normalized EMG over increased velocity (i.e., EMG-change HV–LV) during MG muscle stretches was comparable to previous reported MG data. Specifically, the SCP group showed a median EMG-change of 3.96% (7.26) when comparing muscle activity at HV to LV stretches, which is in line with previously reported median scores of 2.57% (3.25) of children with the same spasticity scores (MAS 1.5) (17). The children in the HSP group presented MAS scores of 2 and normalized EMG-change of 7.88%. Previous research reported similar EMG change scores (median of 7.25%) in a cohort of children with SCP presenting MAS scores of 2.3 (17).

### Clinical Implications

The objective instrumented investigation of MG muscle characteristics in the HSP group may help to gain more insight in the clinical impairments of children with HSP and to reveal potential markers for accurate differential diagnosis to SCP. In line with the findings of Harding et al., our results suggest the potential predominance of hyperreflexia rather than muscle weakness with the latter indirectly indicated based on the muscle volume results (1). Smaller muscle volumes are likely to generate less active force and are consequently indicative for decreased muscle strengths (46, 47). In the HSP group, muscle volumes tended to be less altered in comparison with the SCP group. This observation was combined with a tendency of higher hyperreflexia values, suggesting the predominance of spasticity in HSP. Yet, this trend needs to be confirmed in larger study samples.

Noticeably, it is important to investigate hyperreflexia using an objective instrumented assessment while performing quantitative analysis of the neurophysiological muscle response during passive muscle stretches in order to describe the neural contributor of hyper-resistance. Manual clinical examinations, such as MAS and MTA, are unable to discriminate among neural or non-neural contributors (19, 22, 48). The observed tendency of more hyperreflexia in the HSP group in comparison with rather similar MAS and MTA scores between the HSP and SCP groups confirms this statement.

The occurrence of clonus (i.e., repetitive oscillatory muscle contractions following stretch) is described as an upper motor neuron lesion sign and is generally presented in combination with hyperreflexia (49, 50). In an additional exploration, normalized RMS-EMG graphs of the high velocity stretches were visually inspected for the number of oscillations over the time period for which a minimal RMS-EMG activity was observed. Figure 2 represents an HSP case with a clear clonus, indicating 24 oscillations over a time period of 4 s. Both the results of the clinical examination scores for clonus and number of oscillations over time are presented per participant in Supplementary Table 4. The presence of clonus in particular in the HSP group is equally observed according to these two approaches. Moreover, in the children with HSP showing clinically a quick clonus with no stop, an increasing number of oscillations was suggested in comparison with children with a quick stop. In contrast, in the SCP group, only three children showed a clonus in the clinical examination, whereas the phenomenon was confirmed by EMG data for only two children with SCP. However, more research should be conducted to provide valid and reliable methods to quantify clonus with the described assessment of hyperreflexia in both children with HSP and SCP. In the context of the current study, hyperreflexia and clonus can be suggested as clinical markers to distinguish the two disorders.

Treatment approaches in children with HSP are symptomatic and generally aimed to reduce muscle spasticity. Treatment management mainly involves physiotherapy and tone-reducing medication. However, a recent review highlighted the lack of high-level studies, like randomized control trials, that provide sufficient evidence to promote these treatments in children with HSP (6). Yet, the hyperreflexia data of the current study might indicate tone reduction as an important treatment goal in children with HSP. Despite limited data on the effectiveness of BTX injections on focal spasticity and function in children with HSP (6, 7), the presence of high-velocity-dependent phenotypes indicates the potential positive outcomes of BTX treatment. Indeed, in children with SCP, previous research on the effect of BTX demonstrated more pronounced decreases in muscle tone in children with high-velocity-dependent muscle activation patterns compared with length-dependent muscle activation patterns (21). Apart from daily orthotic management and regular physiotherapy as the general treatment approach, the use of BTX is widely applied as tone-reducing treatment in children with bilateral SCP (51). Since the muscle characteristics were found to be similar, it is reasonable to recommend analogous treatment approaches in children with HSP and SCP.

### Limitations and Future Perspectives

It should be noted that some limitations are present in the current study. The main drawback of this retrospective study is the small sample size, which compromises the power of the statistical tests and may prevent generalization of the observations. Due to the nature of the single-center, retrospective study design, we were limited to the available datasets for each subgroup. In addition, specific inclusion criteria for both the HSP and SCP groups were applied, aiming to reduce the clinical heterogeneous presentation. Despite these efforts, the muscle data presented high variability, although normalization to anthropometric data successfully decreased this heterogeneity. This finding suggests that children of the different cohorts should be matched for anthropometric data rather than for age. Notably, HSP is a rare disease with an estimated prevalence of 1:10,000, and not all case were genetically confirmed (52). The restriction to only include pure HSP types enlarges the rareness of the disease. Future studies should aim for a prospective case-control design and could be carried out as a multi-center study to increase the size of the dataset.

This study only addressed the MG muscle. The restriction to the MG as muscle of interest was fixed due to (a) the available muscle-specific datasets and (b) the ability to compare our results with previous publications. Hamstring muscle spasticity and weakness have been described as clinical impairments in HSP (4, 35). Since muscle activation patterns were found to be muscle specific, future studies should investigate these muscle-specific characteristics. In the current study, the observed high-velocity-dependent pattern of the MG muscle confirmed this previously reported MG-specific pattern (20).

Furthermore, the current study focused on the neurophysiological response to passive muscle stretch. Future studies should further explore the specific contribution of both neural and non-neural components of the hyper-resistance against passive muscle stretch using instrumented assessments in children with HSP. In addition, these assessments should be used to consider the effectiveness of frequently applied tone-reducing treatment approaches in children with HSP.

## Conclusion

This is the first study that explored the MG muscle characteristics in children with HSP using instrumented impairment assessments. Muscle volume deficits, high-velocity hyperreflexia, and ankle clonus were presented in children with HSP. In general, instrumented assessments of the altered muscle morphology and hyperreflexia suggested similar MG muscle-related impairments in pediatric pure HSP types with pediatric onset and in children with bilateral SCP. Hence, the use of analogous treatment approaches might be supported by the study results. Due to the limited statistical power caused by the limitations in sample size, the study results should be confirmed in larger cohorts.

## Data Availability Statement

The datasets generated for this study are available on request to the corresponding author.

## Ethics Statement

The studies involving human participants were reviewed and approved by Ethical Committee of the University Hospitals Leuven and KU Leuven (S56977 and S59945). Written informed consent was provided by the participants' parents/legal guardians.

## Author Contributions

This study was designed by ND, LB-O, EO, KD, and AV. ND, BH, and NP contributed to the data collection of the retrospective database. EO and AV evaluated the eligibility of the participants. ND was responsible for the data processing and conducted all presented analyses. ND, LB-O, KD, and AV contributed to the interpretation of the results and were involved in the critical revision and editing of the manuscript that was written by ND. All authors approved the final version of the manuscript and agreed to be accountable for the content of the work. All authors have had complete access to the study data throughout the study.

## Conflict of Interest

The authors declare that the research was conducted in the absence of any commercial or financial relationships that could be construed as a potential conflict of interest.
