# Supplementary material for: Muscle Characteristics in Pediatric Hereditary Spastic Paraplegia vs. Bilateral Spastic Cerebral Palsy: An Exploratory Study
Source: Front Neurol. 2021 Feb 26;12:635032. doi: 10.3389/fneur.2021.635032 (PMC7952873; doi:10.3389/fneur.2021.635032)
Supplement: Supplementary file 1 [file Data_Sheet_1.pdf]

Supplementary Material

Supplementary Table S1. Results of the diagnostic investigations for each child in the included HSP group

Participant	**Genetic testing**	**Age of onset**	**Family history**	**Neuro-imaging**	**EMG**	**Metabolic testing**
1	+, SPG3A	1 y 6 m	unknown	-	-	NA
2	+, SPG4	3 m	-	-	-	NA
3	+, SPG3A	2 y	+	NA	NA	NA
4	+, SPG56	2 y	+	-	NA	-
5	-, Clinical exome, Nimbelgen V4 panel	3 y 6 m	-	-	-	-
6	-, Direct sequencing REEP1	9 y	+	NA	NA	NA
7	-, Clinical exome, SeqCP EZ Exome V3 panel	4 y	+	-	NA	-
8	-, Clinical exome, Nimbelgen V4 panel	1 y 3 m	-	-	NA	NA
9	-, Clinical exome, Nimbelgen V4 panel	1 y 6 m	-	-	NA	NA

HSP, hereditary spastic paraplegia; EMG. Electromyography; +, positive; -, negative; SPG, spastic paraplegia genes; y, years; m, months; NA, not assessed.

Supplementary Table S2. Treatment details in the HSP and the total SCP group

	**HSP** (n=9)	**SCP** (n=17)
Physiotherapy (min/week)	120.0 (30-240)	100.0 (60-240)
Use of day orthoses, n	Frequently used, n= 3	Frequently used, n= 7
	Not frequently used, n= 0	Not frequently used, n=3
	Insoles, n=1	Insoles, n=3
	Not used, n=5	Not used, n=4
Use of night orthoses, n	Frequently used, n=0	Frequently used, n=6
	Not frequently used, n=4	Not frequently used, n=1
	Not used, n=5	Not used, n=10
Medication, n	Tone reduction, n=2	Tone reduction, n=3
	Others, n=2	Anti-epilepsy n=2
	Not used, n=5	Others, n=2
		Not used, n=10
Treatment history, BTX (n)	0, n=5	0, n=10
	1-3, n=3	1-3, n=1
	>3, n=1	>3, n=6

The amount of physiotherapy is shown as median (minimum-maximum values). The other data presented frequencies per type of treatments. Frequently used indicated ≥ 50% of the day or night whereas not frequently used indicated <50% of the day or night.

HSP, hereditary spastic paraplegia; SCP, spastic cerebral palsy; n, number; BTX, botulinum neurotoxin injections.

Supplementary Table S3. Clinical scores of children in the HSP and the total SCP group

		**HSP** (n=9)	**SCP** (n=17)
FMS,	5m	6 (5-6)	6 (5-6)
	50 m	6 (2-6)	5 (5-6)
	500 m	5 (2-6)	5 (1-6)
Walking ability, n		Normal, >3k, n=5	Normal, >3k, n=9
		>1k-3k, n=2	>1k-3k, n=3
		500m-1k, n=1	500m-1k, n=2
		<500m, n=1	<500m, n=3
Gait speed*, n		Normal, n=0	Normal, n=0
		Slightly reduced, n=4	Slightly reduced, n=4
		Moderately reduced, n=4	Moderately reduced, n=12
Pain during gait, n		Yes, n=2	Yes, n=3
		No, n=7	No, n=14
Bladder & bowel function, n		Normal, n=9	NA
		Abnormal, n=0	

Data of the Functional Mobility Scale (FMS) are shown as median (minimum-maximum values). The other data presented frequencies per clinical assessment. *The gait speed is defined for 8 children in the HSP group and 16 children in the SCP group. Slightly reduced indicated a walking speed of ≥ 5s over 10m whereas moderately reduced indicated a walking speed of ≥ 10s over 10m. *HSP, hereditary spastic paraplegia; SCP, spastic cerebral palsy; n, number; m, meter; k, kilometers.*

Supplementary Table S4. Clonus observation in the HSP and SCP group

**HSP** (n=9)			**SCP** (n=9)		
Patient	*CE*	*Instrumented assessment*	*Patient*	*CE*	*Instrumented assessment*
01	2	5 peaks over 0.8 sec	01	2	0
02	2	4 peaks over 0.9 sec	02	0	0
03	4	29 peaks over 4.2 sec	03	0	0
04	3	3 peaks over 0.5 sec	04	0	10 peaks over 1.6 sec
05	2	2 peaks over 1 sec	05	0	0
06	1	2 peaks over 0.3 sec	06	2	5 peaks over 1.5 sec
07	0	0	07	1	0
08	3	3 peaks over 0.6 sec	08	0	0
09	NA	24 peaks over 4 sec	09	0	0

Individual clonus data per participant in the HSP and SCP group is presented. The following clonus scores are used: 0, no clonus; 1, clonus after multiple stretches; 2, quick clonus, quick stop; 3, quick clonus, slow stop; 4, quick clonus, no stop. Instrumented assessment quantified the clonus by the number of oscillations over the time period for which a minimal of 0.05 % RMS-EMG activity was observed. *HSP, hereditary spastic paraplegia; SCP, spastic cerebral palsy; n, number; NA, not assessed; sec, seconds; CE; clinical examination.*

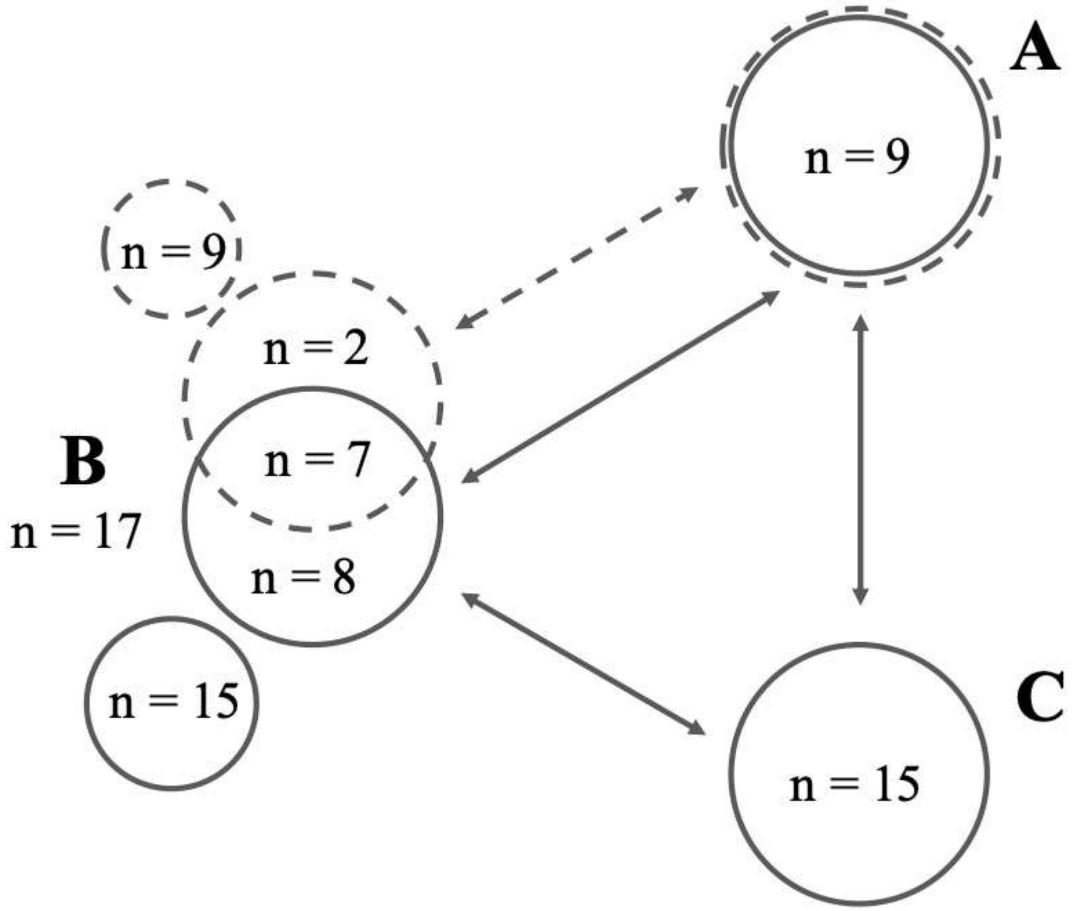

Supplementary Figure 1: Schematic presentation of the included participants (n=41) and related groups: (**A**) children with hereditary spastic paraplegia (n=9), (**B**) children with spastic cerebral palsy (total n=17, subgroups of n=9 and n=15) and (**C**) typically developing children (n=15). The number of children with respect to the group comparisons is indicated for each assessment type: continuous line for 3D ultrasound and dot line for instrumented assessment of hyperreflexia. For seven children in the SCP group, both 3D ultrasound and hyperreflexia data were used.

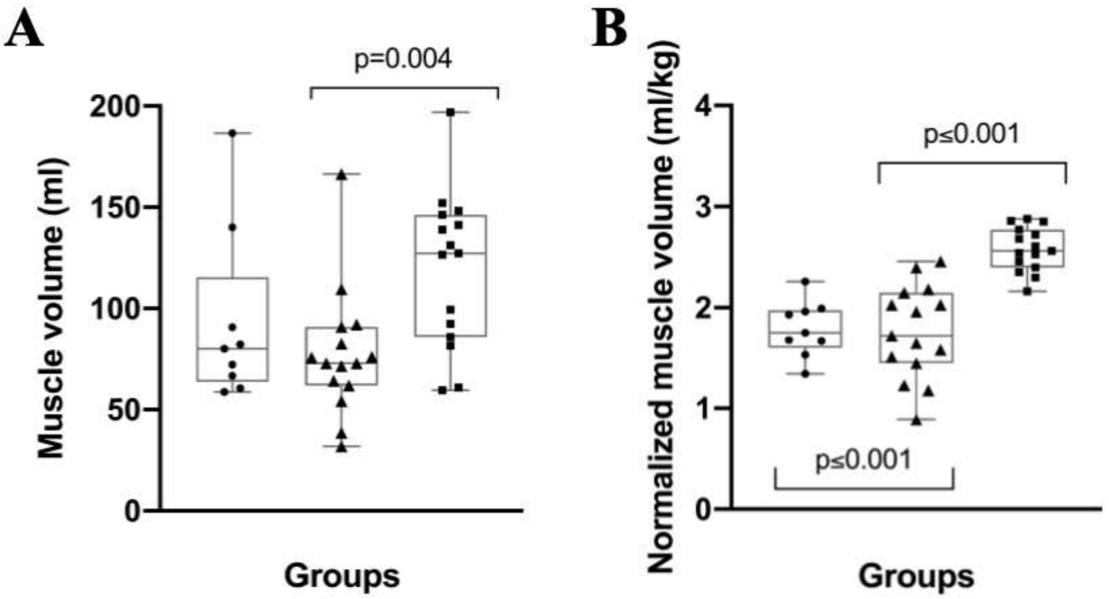

Supplementary Figure 2: Results of **(A)** muscle volume (ml) and **(B)** muscle volume normalized to body mass (ml/kg) with the significant p-values. Individual data points of the different groups are presented with circles for the group of children with hereditary spastic paraplegia, triangles for the children with spastic cerebral palsy and squares for typically developing children.
